# Supplementary material for: Senolytic intervention improves cognition, metabolism, and adiposity in female APPNL−F/NL−F mice
Source: GeroScience. 2024 Aug 9;47(1):1123–38. doi: 10.1007/s11357-024-01308-8 (PMC11872876; doi:10.1007/s11357-024-01308-8)
Supplement: Supplementary file 1 — Supplementary file1 (DOCX 118 KB) [file 11357_2024_1308_MOESM1_ESM.docx]

**Title:** Senolytic Intervention Improves Cognition, Metabolism, and Adiposity in Female APP^NL-F/NL-F^ Mice

**Journal:** *Geroscience*

**Authors:** Authors: Yimin Fang, PhD^1^, Mackenzie R. Peck^1^, Kathleen Quinn^1^, Jenelle E. Chapman^1^, PhD, David Medina, MD^2^, Samuel A. McFadden, MS^1^, Andrzej Bartke, PhD^2,3^, Erin R. Hascup, PhD^1,4^, *Kevin N. Hascup, PhD^1,3,4^

**Corresponding Author:** Kevin N. Hascup, Department of Neurology, Dale and Deborah Smith Center for Alzheimer’s Research and Treatment, Southern Illinois University School of Medicine, Springfield, IL 62794-9268, USA Tel: 217-545-6994, Email: khascup49@siumed.edu


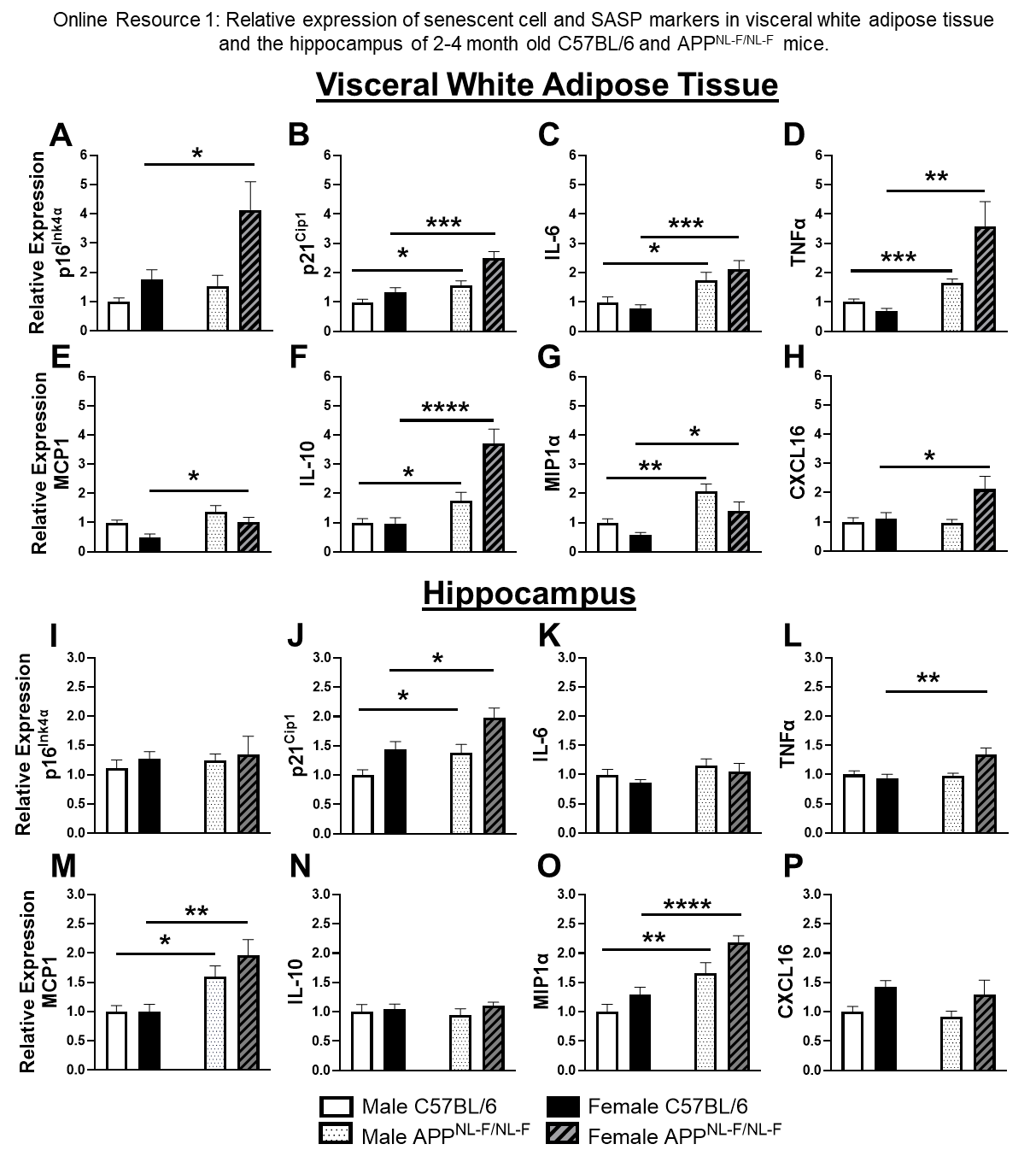


**Online Resource 1 – Relative expression of senescent cell and SASP markers in visceral adipose tissue and hippocampus of four month old C57BL/6 and APP^NL-F/NL-F^ mice.** Gene expression profiles in vWAT (A-H) and hippocampus (I-P). Data are represented as means ± SEM (n=8-12). *p<0.05, **p<0.01, ***p<0.001, ****p<0.0001 based on a two-tailed Student's *t* test.
