# Supplementary material for: Senolytic intervention improves cognition, metabolism, and adiposity in female APPNL−F/NL−F mice
Source: GeroScience. 2024 Aug 9;47(1):1123–38. doi: 10.1007/s11357-024-01308-8 (PMC11872876; doi:10.1007/s11357-024-01308-8)
Supplement: Supplementary file 2 — Supplementary file2 (DOCX 149 KB) [file 11357_2024_1308_MOESM2_ESM.docx]

**Title:** Senolytic Intervention Improves Cognition, Metabolism, and Adiposity in Female APP^NL-F/NL-F^ Mice

**Journal:** *Geroscience*

**Authors:** Authors: Yimin Fang, PhD^1^, Mackenzie R. Peck^1^, Kathleen Quinn^1^, Jenelle E. Chapman^1^, PhD, David Medina, MD^2^, Samuel A. McFadden, MS^1^, Andrzej Bartke, PhD^2,3^, Erin R. Hascup, PhD^1,4^, *Kevin N. Hascup, PhD^1,3,4^

**Corresponding Author:** Kevin N. Hascup, Department of Neurology, Dale and Deborah Smith Center for Alzheimer’s Research and Treatment, Southern Illinois University School of Medicine, Springfield, IL 62794-9268, USA Tel: 217-545-6994, Email: khascup49@siumed.edu

**
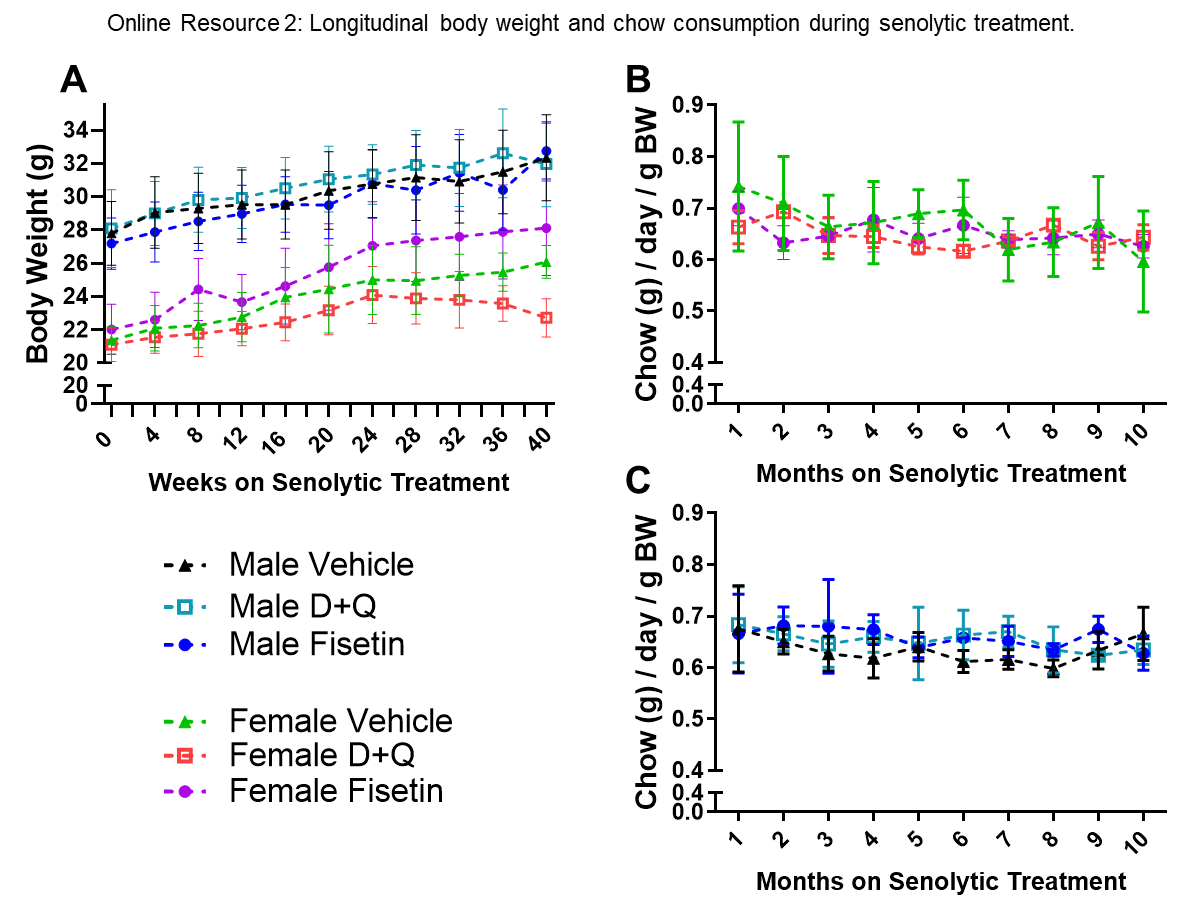
**

**Online Resource 2 – Longitudinal body weight and food consumption during senolytic treatment.** Body weight (BW; A) and chow consumption (B-C) were monitored weekly with the later averaged across treatment months. Time scale denotes start of senolytic treatment when mice were four months of age. Data are represented as means ± SEM (n=5-10).
