# Supplementary material for: Senolytic intervention improves cognition, metabolism, and adiposity in female APPNL−F/NL−F mice
Source: GeroScience. 2024 Aug 9;47(1):1123–38. doi: 10.1007/s11357-024-01308-8 (PMC11872876; doi:10.1007/s11357-024-01308-8)
Supplement: Supplementary file 3 — Supplementary file3 (DOCX 76 KB) [file 11357_2024_1308_MOESM3_ESM.docx]

**Title:** Senolytic Intervention Improves Cognition, Metabolism, and Adiposity in Female APP^NL-F/NL-F^ Mice

**Journal:** *Geroscience*

**Authors:** Authors: Yimin Fang, PhD^1^, Mackenzie R. Peck^1^, Kathleen Quinn^1^, Jenelle E. Chapman^1^, PhD, David Medina, MD^2^, Samuel A. McFadden, MS^1^, Andrzej Bartke, PhD^2,3^, Erin R. Hascup, PhD^1,4^, *Kevin N. Hascup, PhD^1,3,4^

**Corresponding Author:** Kevin N. Hascup, Department of Neurology, Dale and Deborah Smith Center for Alzheimer’s Research and Treatment, Southern Illinois University School of Medicine, Springfield, IL 62794-9268, USA Tel: 217-545-6994, Email: khascup49@siumed.edu


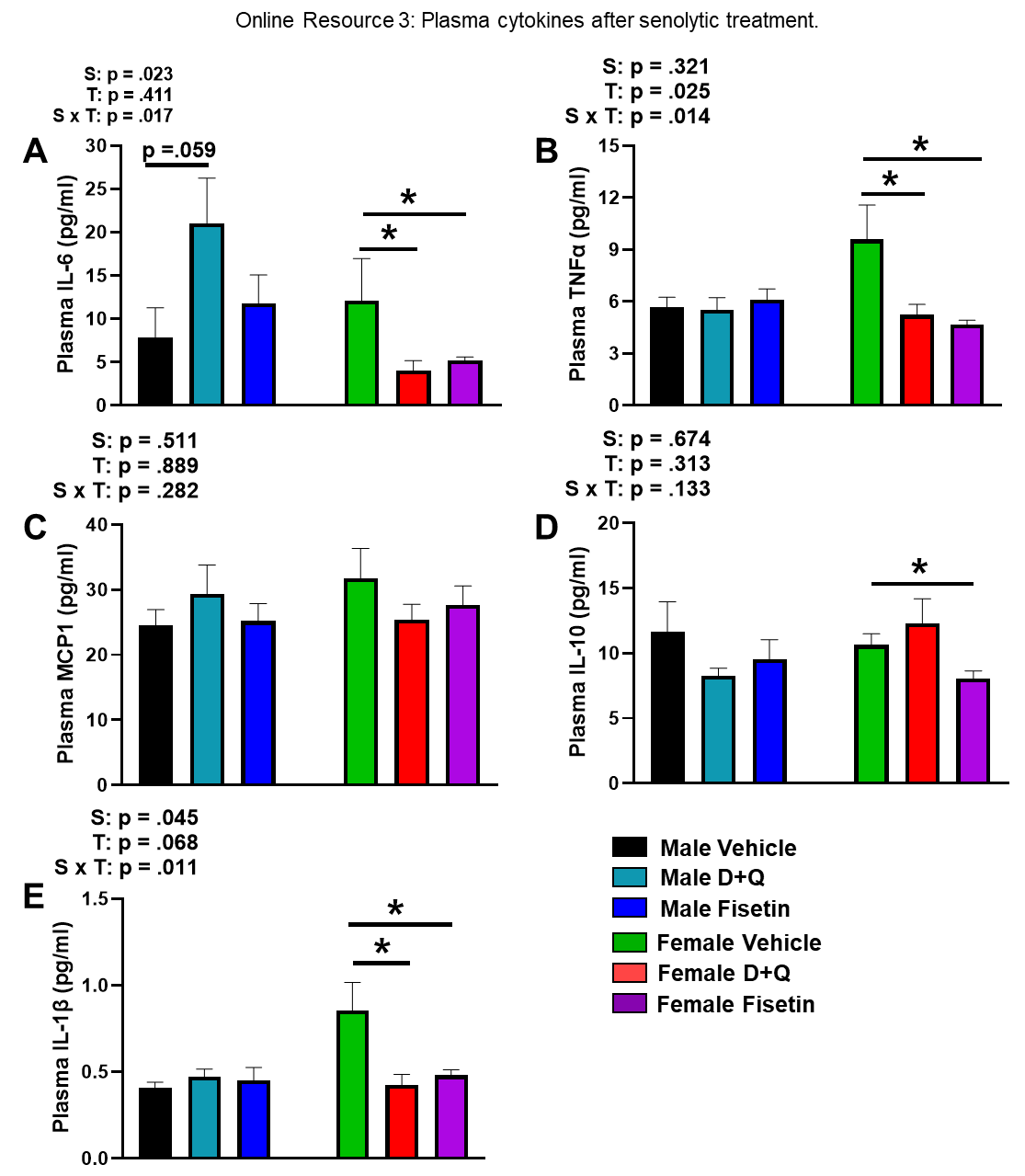


**Online Resource 3 – Plasma cytokines after senolytic treatment.** Circulating plasma IL-6, TNFα, MCP1, IL-10, and IL-1β (A-E) levels in male and female APP^NL-F/NL-F^ mice. Data are represented as means means ± SEM (n=6-10). Results of a two factorial analysis are shown above each bar graph for the Sex (S) and Treatment (T) categorial variables and their interaction (S x T). *p<0.05 based on a two-tailed Student's *t* test.
